# Diversity of RNA Viruses in Declining Mediterranean Forests

**DOI:** 10.3390/microorganisms14071445

**Published:** 2026-06-30

**Authors:** Sergio Diez-Hermano, Alba Diez-Galán, Pedro Luis Pérez-Alonso, Wilson Acosta Morel, Jonatan Niño-Sanchez, Marcos de la Peña, Julio Javier Díez

**Affiliations:** 1 Department of Plant Production and Forest Resources, Sustainable Forest Management Research Institute (iuFOR), Higher Technical School of Agricultural Engineering (ETSIIAA), University of Valladolid, 34071 Palencia, Spain; sergio.diez.hermano@uva.es (S.D.-H.); wilson.acosta@uva.es (W.A.M.); jonatan.nino@uva.es (J.N.-S.); 2 One Health Chair On Forest and Human Health, University of Valladolid, 34071 Valladolid, Spain; 3 Institute for Plant Molecular and Cellular Biology (CSIC-UPV), 46022 Valencia, Spain; rivero@ibmcp.upv.es

**Keywords:** forest pathology, metavirome, virome, virosphere, *Castanea*, *Pinus*, *Quercus*

## Abstract

Global change alters forestry habitats and facilitates the entry of new pathogens that do not share a co-evolution history with the forest, leading them into a spiral of decline. As a result, relationships between forest organisms become imbalanced. RNA viruses are of particular concern given their capacity to infect hosts across different kingdoms of life, yet the viromes of Mediterranean forests remain largely unexplored. For this reason, the study of RNA viruses is essential for understanding how viral flow across different hosts might occur, and to prevent possible outbreaks of diseases in the future. In this work, the RNA virus diversity found in trees, arthropods, and fungi from declining Mediterranean forests is described. To this extent, three habitats (*Quercus ilex*, *Castanea sativa* and *Pinus radiata*) were sampled and RNAseq was performed on tree tissues, arthropods, and fungi. In total, 146 viral sequences were detected by searching for matches with conserved motifs of the RNA-dependent RNA polymerase (RdRP) using Palmscan. Up to 15 viral families were identified, with *Botourmiaviridae* (28.7%) and *Partitiviridae* (9.6%) being the most abundant. In terms of genome type, ssRNA(+) viruses were the most represented (83.5%), followed by dsRNA (15%) and two ssRNA(−) representatives. Notably, families with documented cross-kingdom capabilities such as *Hypoviridae* (1), *Mitoviridae* (6), and *Narnaviridae* (5) were detected across multiple host types, with one *Mitovirus* sequence recovered unexpectedly from pine tree tissue. Distribution of viruses across ecosystems included *Q. ilex* (57.5%), *P. radiata* (26.7%), and *C. sativa* (15.8%). Interestingly, two RdRP sequences showed no similarity to any entry in current viral databases, representing potentially novel viruses warranting further characterization. These findings reveal a rich and partially uncharacterized RNA virosphere in declining Mediterranean forests and underscore the importance of multi-host virome surveys for understanding viral flow across kingdoms in threatened ecosystems.

## 1. Introduction

In recent decades, it has become increasingly evident that all biotic components of forest ecosystems (bacteria, protists, fungi, vertebrate, and invertebrate animals) host their own viral communities (viromes), which together form the metavirome or virosphere of the forest ecosystem. Recent advances in metagenomics and artificial intelligence have greatly expanded the understanding of this hidden diversity. A deep learning model has identified over 160,000 potential RNA virus species grouped into approximately 180 supergroups globally [1]. Large-scale environmental studies have also uncovered thousands of new RNA viruses in terrestrial and sediment environments, including Mediterranean-like soils [2]. Despite this progress, the viromes of forest ecosystems remain among the least characterized, particularly in the context of ecosystems under anthropogenic and climatic stress.

Currently, around 64 virus species are known to affect 19 forest plant species in temperate and urban forests [3], although this figure is likely to be an underestimate given the rapid expansion of virome studies. Most belong to the (+)ssRNA genome group, including families such as *Betaviridae*, *Secoviridae*, *Bromoviridae*, *Tombusviridae*, *Virgaviridae*, and *Mayoviridae*. The (−)ssRNA component of forest plant viromes has proven more diverse than initially recognized, with members of *Fimoviridae* (which replaced the former *Emaraviridae* and includes the genus *Emaravirus*), *Rhabdoviridae*, *Phenuiviridae*, *Tospoviridae*, and *Aspiviridae* all reported infecting woody and forest plant species [4,5]. A few dsRNA viruses have also been described in pine and ash, as well as some dsDNA viruses with reverse transcriptase activity in *Betula* sp., *Castanea sativa*, and *Fraxinus americana*. Among the pathogenic genera, emaraviruses and badnaviruses are considered causative agents of disease in trees. Most viruses affecting forest fungi and oomycetes have a dsRNA genome and belong to the *Partitiviridae*, *Totiviridae*, *Curvulaviridae*, and *Reoviridae* families. (+)ssRNA viruses are classified within *Hypoviridae*, *Endornaviridae*, *Botourmiaviridae*, *Fusariviridae*, and *Mitoviridae*. Several of these have been shown to modulate the pathogenicity of important forest pathogens, such as *Cryphonectria parasitica*, *Ophiostoma novo-ulmi*, *Gremmeniella abietina*, *Fusarium circinatum*, *Heterobasidion annosum*, and *Hymenoscyphus fraxineus* [6].

Recent studies have expanded this knowledge, revealing novel dsRNA and ambisense viruses in pathogens such as *Armillaria* spp. and *C. parasitica* [7]. A comprehensive virome survey of *Armillaria* isolates from different regions uncovered unexpected (+)ssRNA and circular ambisense viruses [8]. Furthermore, analysis of public sequencing data identified 12 novel fungal RNA virus sequences, revealing the existence of an unexplored viral dark matter [9].

Cross-kingdom viruses play a significant role in forest ecosystems, particularly between trees, fungi, and insects [10]. Some *Toti*-, *Partitiviridae*, *Endornaviridae*, and *Chrysoviridae* members replicate in meristematic cells in plant and can infect fungi in vitro [11]. Natural cross-kingdom infection has been experimentally demonstrated, such as in *Rhizoctonia solani*, which acquired and transmitted cucumber mosaic virus [12]. Functional cooperation between plant and fungal viruses has also been documented, facilitating transmission across vegetatively incompatible fungal strains [13]. Notably, a negative-sense RNA virus (Valsa mali negative-strand RNA virus 1, VmNSRV1) was recently shown to naturally infect both a plant host (apple) and its fungal pathogen (*Valsa mali*), providing new evidence of interkingdom infection [14].

Despite their importance as vectors, insect viromes in forest ecosystems remain poorly characterized. Once inside the insect, plant viruses can develop forms of non-persistent transmission (in external structures of the insect) or circulative transmission (using internal compartments as reservoirs). In some cases, circular transmission may involve replication of the plant virus within the cells of the insect host [15]. Recent research on three wheat aphid species identified 12 new RNA viruses and 90 endogenous viral elements (EVEs), suggesting complex viral interactions involving potential fungal associations [16]. Viruses have also been found in lepidopterans (*Lymantria dispar*, *Thaumetopoea pityocampa*, *Leucoma salicis*), coleopterans (*Ips typographus*), and hymenopterans (*Neodiprion sertifer*) [17,18]. Importantly, arthropods may act as bridges for viral transmission between plant and fungal hosts, yet this role has rarely been investigated in forest virome studies through simultaneous sampling of multiple host types.

Mediterranean forests are among the terrestrial ecosystems most threatened by globalization and climate change [19]. Predicted reductions in precipitation and rising temperatures will expose these ecosystems to prolonged droughts and more frequent wildfires [20]. These stresses decrease the resilience of Mediterranean tree species against pests and diseases, facilitating the invasion of novel pathogens. Recent surveys of *Rosellinia necatrix*, a soilborne fungus widespread in Mediterranean areas, reported a virosis incidence of ~14% in isolates from Spain, Israel, and Italy, revealing viruses from *Partitiviridae*, *Hypoviridae*, *Megabirnaviridae*, and *Fusagraviridae* families, and new *Megatotiviridae* members [21].

RNA viruses are known agents of emerging infectious diseases and may be released following forest disturbances [22,23], increasing the risk of unexpected pandemics. In this context, *Quercus ilex*, *Castanea sativa*, and *Pinus radiata* represent three of the most ecologically and economically relevant tree species in the Iberian Peninsula, all of which are currently experiencing significant decline associated with drought stress, fungal pathogens and insect pests, making them priority targets for virome characterization.

Given the ability of RNA viruses to cross barriers and alter pathogenicity, it is essential to better understand viral flows in endangered ecosystems. Here, the metavirome of three declining Mediterranean forests is described using high-throughput sequencing, analyzing viral diversity across plants, fungi, and arthropods.

## 2. Materials and Methods

### 2.1. Sampling Sites and Procedure

**Figure 1:**
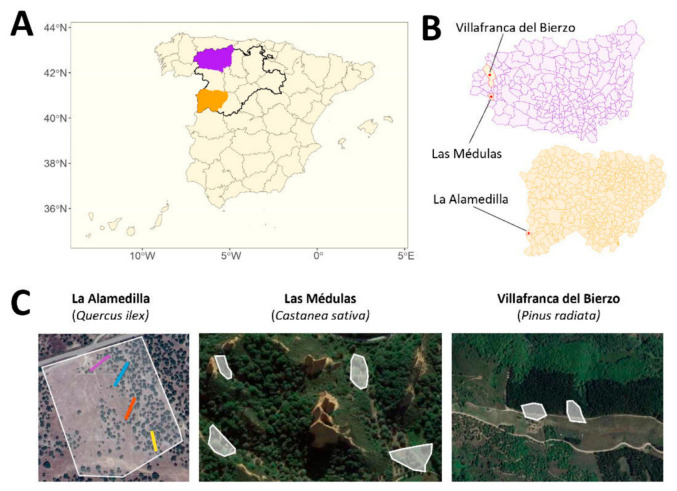
Map of sampling sites. (**A**)—Map of Spain. Castile and Leon region is highlighted with a black and thick contour. Orange and purple areas correspond to Salamanca and León provinces, respectively. (**B**)—Municipalities from where samples were taken (top: León, bottom: Salamanca). (**C**)—Sampling areas (white polygons) overlayed on top of physical maps. Colored lines in La Alamedilla correspond to four sampling transects.

Bark, wood, and leaf samples were extracted from trees of *Castanea sativa* (chestnut), *Quercus ilex* (holm oak), and *Pinus radiata* (Monterrey pine) from Castile and Leon (Spain) forests (Table 1 and Figure 1). Each sampling site corresponded to a single tree species. Sampling was conducted for all four sites in June and July 2021. Trees samples were selected based on symptoms associated with forest decline and disease. *P. radiata* individuals exhibited resinous cankers and dieback symptoms, *Q. ilex* trees showed decline symptoms, and *C. sativa* individuals presented chestnut cankers. Material was sampled from sixteen trees per plot and pooled. After removing the external bark, one sample was taken per tree from the main trunk at a height of 50 cm over the collar, to a depth of 2–3 cm. Only xylem and the internal bark layer (phloem) were considered in the analysis. Living individuals of common arthropods (ants; Formicidae, grasshoppers; Caelifera, and beetles; Coleoptera) were also collected (8–10 specimens each). Samples were stored at 4 °C for one day prior to processing and at −80 °C afterwards. Arthropods were placed in collection tubes and transported to the laboratory, where they were stored in ethanol until RNA extraction.

### 2.2. Sample Processing and Sequencing

Fungi were cultured from the tree and arthropod samples. Bark, wood, and leaves were cut into small pieces and placed on Petri dishes with PDA+ampicillin (100 μg/mL) medium for 3 days at 25 °C. Arthropods were washed in a Ringer solution and 50 μL of the solution were poured and spread on Petri dishes with PDA+ampicillin medium for 3 days at 25 °C, which is likely to have favored fast-growing fungi and may have led to an underestimate of total fungal diversity. Visually different fungal colonies were picked, isolated, and stored at −80 °C.

Frozen samples of bark, wood, leaves, and arthropods were pulverized and pooled per sampling site prior to RNA extraction. Isolated fungi were pooled likewise. Total RNA was purified from trees, arthropods, and fungi using a Spectrum Plant kit (Sigma Aldrich, St. Louis, MO, USA) following the manufacturer’s protocol. Quality control with Qubit yielded RIN values > 7 for all extractions. Samples were sent to Macrogen for sequencing (Illumina Miseq with TruSeq libraries and ribosomal RNA depletion, 150 bp paired-end reads (Seoul, Republic of Korea).

### 2.3. Bioinformatic Analysis

Raw reads were cleaned and trimmed using Cutadapt v.3.5 (−q 20) [24]. Contigs were assembled with SPAdes v.3.15.5 (rnaviralSPAdes.py) [25] and scanned for the presence of RNA-dependent RNA polymerase (RdRP) sequences using Palmscan v1.0 [26], a software that searches sequencing data for matches with the conserved motifs A, B, and C (the “palmprint”) from the primary protein sequence of RdRP. RdRP sequences that matched any non-viral organism in the nr NCBI database were discarded. Viral identities were assigned to the remaining RdRPs by aligning the found sequences against PALMdb [26] and RdRp-scan [27] databases using DIAMOND v2.1.8 [28]. Taxonomy was assigned using percent identity thresholds established for RNA viruses (species at >90%, genus at >70% and family at >30%), following criteria previously applied in environmental virome studies [26,27]. Multiple sequence alignment was performed with MAFFT (E-INS iterative refinement method) [29] and used as input for phylogenetic tree building in IQ-TREE (default parameters) [30] with ultrafast bootstrap [31] and automatic model selection [32].

Viral diversity analyses were performed and visualized in R v.4.2.1 [33] using the packages ggtree v.4.0.5 [34], ggmsa v.1.16.0 [35], and seqinr v.4.2-36 [36]. Sequence logos were plotted with Skylign [37] (https://skylign.org; accessed on 19 January 2026).

## 3. Results

### 3.1. Taxonomy and Distribution of RdRPs

**Figure 2:**
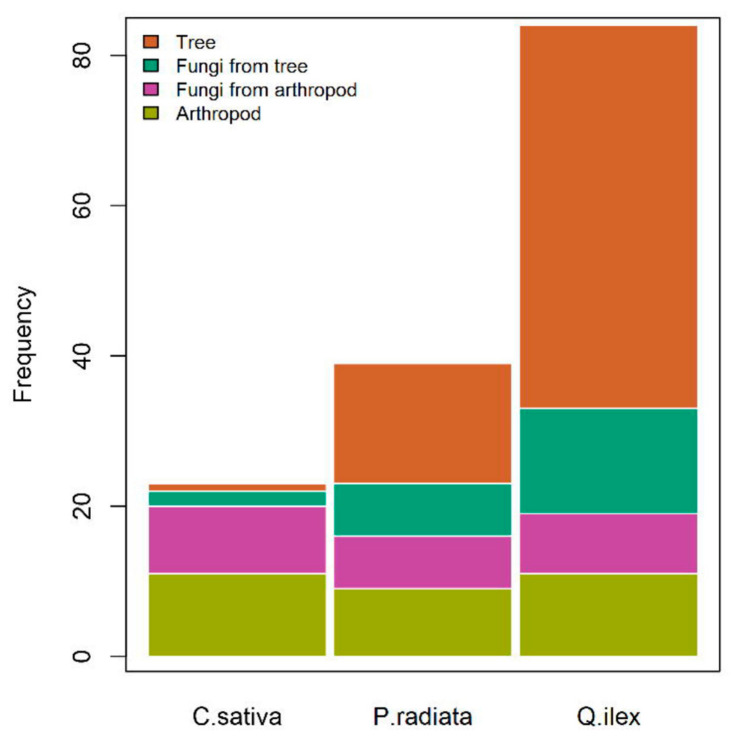
RdRP counts by habitat and sample type.

A total of 146 RdRPs were identified across all analyzed samples, from an equivalent sampling effort across the three habitats (sixteen trees and associated arthropods and fungi per plot). Of these, 122 (83%) were assigned to existing viral groups with different levels of taxonomic resolution, 22 (15%) had matches in current databases but lacked taxonomic assignment, and 2 were absent from any database (Table 2 and Table 3). Most of the RdRPs were found in the habitat of *Q. ilex* (57.5%), followed by *P. radiata* (26.7%) and *C. sativa* (15.8%) (Figure 2 and Table 4), suggesting that holm oak forests harbor a substantially richer RNA virosphere than the other two ecosystems sampled. Regarding sample type, nearly half of the RdRPs were found in tree samples (46.5%), followed by arthropods (21.2%), fungi from arthropods (16.4%), and fungi from trees (15.7%). Notably, one RdRP sequence assigned to the genus *Mitovirus* (family *Mitoviridae*) was recovered from pine tree tissue, a host type outside the known fungal host range of this genus, representing a potentially anomalous host association warranting further investigation.

**Figure 3:**
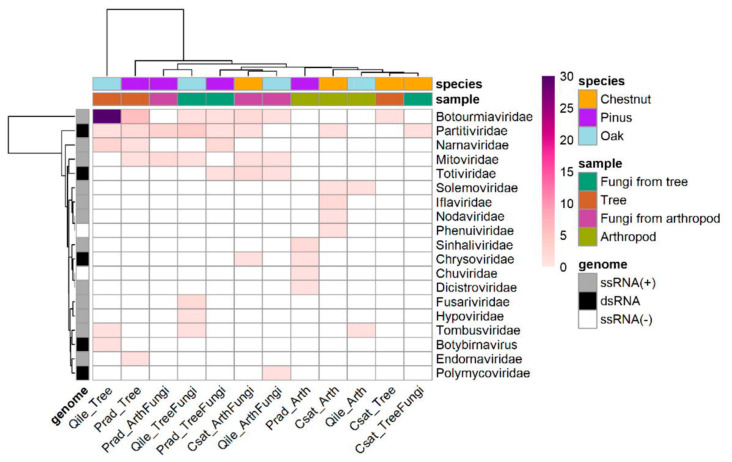
Distribution of viral families per habitat, sample type, and genome type. Color gradient from white to purple indicates abundance.

The most numerous viral families were *Botourmiaviridae* (28.7%), *Partitiviridae* (9.6%), *Mitoviridae* (4.1%), and *Narnaviridae* (4.1%) (Figure 3). In terms of genome type, ssRNA(+) viruses were the most represented (83.5%), followed by dsRNA (15%) and two ssRNA(−) representatives, *Chuviridae* and *Phenuiviridae*, found in arthropods from pine and chestnut habitats, respectively.

The distribution of RdRPs revealed that sample types have different viral profiles (Figure 3). Some families were exclusive to fungi (*Totiviridae*, *Fusariviridae*, *Hypoviridae* and *Polymycoviridae*) or arthropods (only in chestnut habitat: *Iflaviridae*, *Nodaviridae*, *Phenuiviridae*; and only in pine habitat: *Sinhaliviridae*, *Chuviridae*, and *Dicistroviridae*). One family was found to be exclusive to holm oak habitat (*Tombusviridae*), regardless of sample type. *Endornaviridae* family was found only in pines, whereas *Botybirnavirus* was exclusive to holm oaks.

### 3.2. Phylogeny of RdRPs

**Figure 4:**
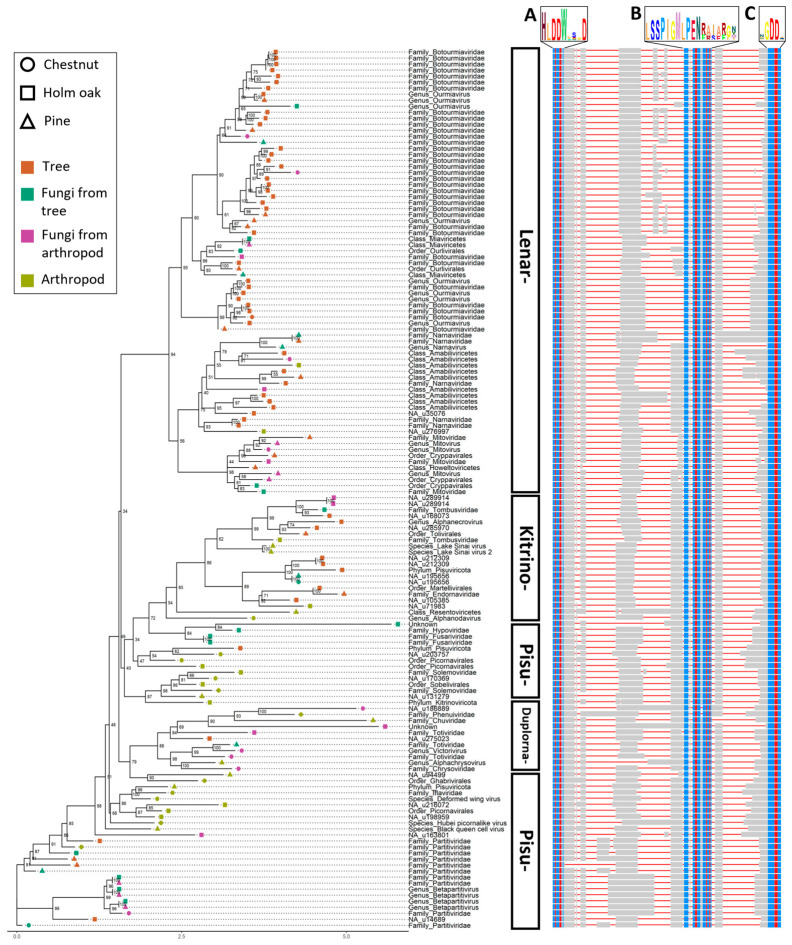
Phylogenetic tree of RdRPs. Left: consensus tree with bootstrap confidence values. Each branch is labelled with the taxonomic level followed by the identity assigned to that particular RdRP. Vertical rectangles with black outline indicate viral phyla (−viricota). Right: multiple sequence alignment with A, B, and C motifs and their corresponding logos. Blue and red regions indicate well-conserved residues.

MSA of the RdRPs resulted in the dendrogram shown in Figure 4. RdRPs from trees and arthropod samples clustered in two distinct groups, while RdRPs from isolated fungi were interspersed, forming heterogeneous subgroups. No grouping by habitat was observed except for most RdRPs from holm oak samples clustering together within the *Lenarviricota* supernode. All RdRPs identified to species level belonged to arthropod samples: Lake Sinai virus 1 and 2, deformed wing virus, black queen cell virus, and Hubei picorna-like virus 15, in the *Kitrinoviricota* and *Pisuviricota* nodes. In nodes where RdRPs from tree fungi and arthropod fungi were paired, as was the case for some *Betapartitivirus* and *Narnaviridae* (bottom part of the tree), their sequences shared 100% identity. In all other cases, the homology in the conserved RdRP motifs A, B, and C was at least greater than 50%.

RdRPs existing in current databases but lacking taxonomy assignment (NA in Figure 4) were classified as *Kitrinoviricota*, *Duplornaviricota*, or *Pisuviricota*. As for the unknown RdRPs (with bona fide A, B, and C motifs but absent from databases), one shared high sequence similarity with the *Hypoviridae* representative and was found in the same sample type (fungi from holm oaks), whereas the other was located in the vicinity of ssRNA(−) viruses (*Chuviridae* and *Phenuiviridae*) and belonged to *Negarnaviricota*, sharing no common origin with the former.

## 4. Discussion

The study of environmental viromes has grown in popularity thanks to recent major advances in massive sequencing technologies. The scale of these analyses is highly variable, with some studies focusing on a particular species [38,39], complete ecosystems [40], or different environments [41,42]. However, most of the advances in plant virology focus on crops or fruit trees of economic interest, which contrasts with the scarcity of such studies in forestry [3]. Reducing the scope even further to Mediterranean forests, with the Mediterranean basin being considered a biodiversity hotspot [43], hardly any information is available. In the present work, focused on three Mediterranean forest habitats in north-western Spain, 146 RdRPs of viral origin have been found, of which 22 have no clear taxonomy yet and 2 are completely unknown. These findings highlight the great lack of knowledge about viral diversity that exists in Mediterranean forest ecosystems.

Most of the reported RdRPs were identified as ssRNA(+) viruses, which are accepted to be the evolutionary origin of the other two identified groups, ssRNA(−) and dsRNA [44,45]. The holm oak habitat showed the highest viral richness (57.5% of all the RdRPs). Holm oak samples were taken in a “dehesa”, a unique ecosystem in the Iberian Peninsula that has been modified by man to simultaneously obtain livestock and non-timber forest resources. This makes dehesas ecosystems with particularly high biodiversity [46,47], so it is not surprising that there is also a high viral diversity that has never been investigated.

Some of the viral families were found to be ubiquitous across sample types and habitats, which could be interpreted as a first indication of their cross-kingdom potential. This is the case for *Botourmiaviridae*, *Partitiviridae*, and *Narnaviridae*. Many genera in *Botourmiaviridae* family are known to infect fungi, and some of them have been identified on grapevine leaves affected by the oomycete *Plasmopara viticola* [48]. Mycoviruses of this family can persist in their fungal host without the need for a capsid and are thought to require only the RdRP to replicate [49]. Only the genus *Ourmiavirus* infects plants exclusively [50]. The RdRP of this genus is most similar to that of the genera *Mitovirus* and *Narnavirus* (family *Narnaviridae*) and its movement protein (MP) is similar to that of the family *Tombusviridae*. These viruses are very easily transmitted mechanically, and no vector has been identified, so horizontal transmission is thought to be possible [51].

The *Narnaviridae* family has the simplest genome of all RNA viruses, encoding only one polypeptide in which RdRP is found. Within this family, the genus *Narnavirus* replicates in the cytoplasm, while *Mitovirus* replicate in mitochondria and can cause hypovirulence in pathogenic fungi [52,53]. Although no *Mitovirus* have been found outside fungi, one RdRP identified as a *Mitovirus* was found in a pine tree sample in the present study. Nonetheless, *Mitovirus* sequences have previously been found in plant mitochondrial sequences [54,55]. As for the *Partitiviridae* family, many of its genera have characteristic hosts, which can be plants (*Deltapartitivirus*), fungi (*Gammapartitivirus*), or protozoa (*Cryspovirus*). Many sequences of this family have also been identified in arthropods [56]. One genus, *Betapartitivirus*, whose hosts can be plants or fungi, was identified. Regarding the family *Tombusviridae*, this has been described in animals [57], plants [58], and fungi [59], consistent with the detection of this family in all samples types, although restricted to holm oak habitat. *Tombusviridae* infections are frequently confined to the root system, but they can infect the whole plant thanks to their MPs [60]. The *Solemoviridae* family infects plants, but it is known that one of its modes of transmission is also the use of insects as vectors [61], which may explain the detection of this family exclusively in arthropods.

For some families, only one RdRP was detected in the samples analyzed, but evidence of their ability to infect several kingdoms has been described. This is the case for *Totiviridae*, *Chuviridae*, *Phenuiviridae*, *Chrysoviridae*, *Polymycoviridae*, *Endornaviridae*, and two riboviruses with incomplete classification: the genus *Botybirnavirus* and the species Hubei picorna-like virus 15 (unclassified ribovirus). The family *Totiviridae* is associated with latent infections in fungi and protozoa. The families *Chuviridae* and *Phenuiviridae* were the only ones detected in this study possessing a ssRNA(−) genome. Many endogenous viral elements inserted within the host genome have been related to *Chuviridae* infections [62]. The *Chrysoviridae* family infects fungi, plants, and insects [63]. The *Polymycoviridae* family affects fungi, in which it causes hypovirulence or hypersensitivity to antifungals or bacteria, and is even capable of modulating carbon, nitrogen, and iron metabolism [63]. *Endornaviridae* are ssRNA(+) viruses that do not have a true capsid; the genome is encapsulated together with a viral replicase and are able to infect plants and fungi. The genus *Botybirnavirus* (*Orthornavirae*) has fungi as natural hosts [64]. However, a *Botybirnavirus* RdRP sequence was detected in a holm oak tree sample. This finding most probably reflects the presence of fungal material associated with the sampled tissue, although other explanations, such as fungal colonization or the presence of fungal mycelium within the sample, cannot be excluded. Further studies, including transmission assays and experimental inoculation of both fungal and plant hosts, are required to determine whether cross-kingdom transmission occurs.

Finally, some limitations of the present study should be noted. First, fungal isolation relied on a single culture medium and incubation condition, which may have introduced a selection bias toward fast-growing culturable fungi and excluded part of the naturally associated fungal community. Additionally, many sequences in viral databases have no or incomplete taxonomic assignment, and the high genetic variation of viruses makes it difficult to obtain an accurate assignment at genus or species level. Recurrent updating of the RdRP databases is necessary to facilitate the assignment of new ones. Secondly, although some of the virus families described may be “cross-kingdom” because they contain members that infect several kingdoms, it is more common for individual viruses to specialize in hosts of a single kingdom, as is the case, for example, with the *Totiviridae* family, where some genera infect fungi and others protozoa. Similarly, the in silico analysis performed did not allow verification of whether the detection of RdRPs in organisms from different kingdoms than expected implies that the host is susceptible to the virus, or that it only acts as a vector or asymptomatic carrier.

## 5. Conclusions

This study provides the first comprehensive description of the RNA virosphere of declining Mediterranean forest ecosystems across trees, fungi, and arthropods. A total of 146 RdRP sequences spanning 15 viral families were identified, with *Botourmiaviridae* and *Partitiviridae* being the most abundant and ssRNA(+) viruses the most represented genome type. Viral richness was highest in the *Q. ilex* habitat, reflecting the exceptional biodiversity of the dehesa ecosystem. Several families showed distribution patterns consistent with cross-kingdom potential, and anomalous host associations (including a *Mitovirus* sequence in pine tree tissue) suggest that viral flow across kingdom boundaries may be more frequent than currently recognized. Two RdRP sequences absent from all known databases represent potentially novel viral lineages warranting further investigation. These findings underscore the importance of multi-host metagenomic approaches for understanding viral diversity and dynamics in threatened forest ecosystems facing increasing pressure from climate change and emerging pathogens.

## Figures and Tables

**Table 1:** Description of sampling sites.

Location	Province	Characteristics	Sampled Species
La Alamedilla	Salamanca	Elevation: 756 m Rainfall: 600–854 mm	*Quercus ilex*
Las Médulas	León	Elevation: 511 m Rainfall: 400–500 mm	*Castanea sativa*
Villafranca del Bierzo	León	Elevation: 505 m Rainfall: 400–500 mm	*Pinus radiata*

**Table 2:** Taxonomic distribution of RdRPs.

Phylum	Class	Order	Family	Genus	Species	Not Assigned ^1^	Unknown ^2^
4	14	12	67	20	5	22	2

^1^ RdRPs found in databases that had no taxonomy assigned yet. ^2^ RdRPs that could not be found in any database.

**Table 3:** Taxonomic assignment of RdRPs per genome type.

	ssRNA(+)	ssRNA(−)	dsRNA
Family (-viridae)	Botourmia- Dicistro- Endorna- Fusari- Hypo- Ifla- Mito- Narna- Sinhali- Solemo- Tombus-	Chu- Phenui-	Chryso- Partiti- Polymyco- Toti-
Genus (-virus)	Alphanoda- Alphanecro- Mito- Narna- Ourmia-		Alphacryso- Betapartiti- Botybirna- Victori-
Virus detected	Black queen cell virus Deformed wing virus Lake Sinai virus Lake Sinai virus 2 Hubei picorna-like virus 15		

**Table 4:** Count of RdRPs per habitat and sample type.

	Tree	Fungi from Tree	Arthropod	Fungi from Arthropod
Chestnut	1	2	11	9
Pine	16	7	9	7
Holm oak	51	14	11	8

## Data Availability

Data and scripts used in this study are available in the following GitHub repository: https://github.com/serbiodh/2023_Virome_MedForests accessed on 15 June 2023. Raw sequencing data are available at NCBI SRA under BioProject PRJNA1032577.

## Abbreviations

The following abbreviations are used in this manuscript:

MPs	Movement proteins
PDA	Potato dextrose agar
RdRP	RNA-dependent RNA polymerase
RNA	Ribonucleic acid
ssRNA	Single-stranded ribonucleic acid
